# Development of a machine learning-based signature utilizing inflammatory response genes for predicting prognosis and immune microenvironment in ovarian cancer

**DOI:** 10.1515/med-2023-0734

**Published:** 2023-06-02

**Authors:** Li Dong, Ya-ping Qian, Shu-xiu Li, Hao Pan

**Affiliations:** Department of Obstetrics and Gynaecology, Changzhou Geriatric Hospital Affiliated to Soochow University, Changzhou, No. 7 People’s Hospital, Changzhou, China; Department of Cardiology, The Affiliated Changzhou, No. 2 People’s Hospital of Nanjing Medical University, Changzhou, China

**Keywords:** ovarian cancer, inflammatory response, machine learning, prognosis, CXCL10

## Abstract

Ovarian cancer (OC) represents a significant health challenge, characterized by a particularly unfavorable prognosis for affected women. Accumulating evidence supports the notion that inflammation-related factors impacting the normal ovarian epithelium may contribute to the development of OC. However, the precise role of inflammatory response-related genes (IRRGs) in OC remains largely unknown. To address this gap, we performed an integration of mRNA expression profiles from 7 cohorts and conducted univariate Cox regression analysis to screen 26 IRRGs. By utilizing these IRRGs, we categorized patients into subtypes exhibiting diverse inflammatory responses, with subtype B displaying the most prominent immune infiltration. Notably, the elevated abundance of Treg cells within subtype B contributed to immune suppression, resulting in an unfavorable prognosis for these patients. Furthermore, we validated the distribution ratios of stromal cells, inflammatory cells, and tumor cells using whole-slide digitized histological slides. We also elucidated differences in the activation of biological pathways among subtypes. In addition, machine learning algorithms were employed to predict the likelihood of survival in OC patients based on the expression of prognostic IRRGs. Through rigorous testing of over 100 combinations, we identified CXCL10 as a crucial IRRG. Single-cell analysis and vitro experiments further confirmed the potential secretion of CXCL10 by macrophages and its involvement in lymphangiogenesis within the tumor microenvironment. Overall, the study provides new insights into the role of IRRGs in OC and may have important implications for the development of novel therapeutic approaches.

## 1 Introduction

Ovarian cancer (OC) is a prevalent malignancy among women, characterized by a poor prognosis with the lowest survival rate among gynecologic cancers. In 2020 alone, around 320,000 women worldwide were diagnosed with OC [1]. However, the asymptomatic nature of most OC cases leads to distant metastases at the time of diagnosis [2]. While postoperative chemotherapy and maintenance therapy have shown improved therapeutic outcomes, common side effects such as nausea and vomiting pose a significant challenge [3]. Thus, developing a robust model for predicting OC prognosis is crucial for personalized treatment.

The association between inflammation and cancer has two pathways: an extrinsic path that increases cancer risk due to inflammatory conditions and an intrinsic path that causes inflammation and tumor formation due to genetic alterations [4]. Inflammatory cells and mediators in the tumor microenvironment (TME) orchestrate pro-inflammatory responses that act in an autocrine and paracrine manner on malignant and non-malignant cells [5,6]. Furthermore, recent studies have established a connection between inflammation of the normal ovarian epithelium and an increased risk of OC, such as ovulation, endometriosis, and pelvic inflammatory disease [7]. Inflammatory mediators and cytokines, such as TNF-α, IL-1β, and IL-6, have been implicated in promoting OC growth, progression, and development [8,9] While previous studies have investigated mRNA-level prognostic signatures in OC patients [10,11,12], genetic signatures associated with inflammatory response, TME, and drug sensitivity have not been studied in the field of OC to date.

This study aimed to comprehensively investigate the molecular alterations and clinical relevance of inflammatory response-related genes (IRRGs) in OC. To achieve this, we constructed molecular subtypes, screened hub IRRGs, and established prognostic models. The results of this study provide novel insights into the prognosis of OC and offer new opportunities for personalized treatment strategies.

## 2 Materials and methods

### 2.1 Datasets and data preprocessing

We selected 200 IRRGs from previously published references [13–15]. We then obtained gene expression profile data from the Cancer Genome Atlas (TCGA) [16] and the Gene Expression Omnibus (GEO) [17] databases, including GSE19829, GSE18520, GSE9891, GSE26193, GSE30161, and GSE63885 based on microarray, and the TCGA-OV cohort based on RNA-seq, using the same inclusion criteria. For more detailed description, see Table A1. Only samples with both sequencing data, clinical information, prognostic data, and no duplicate sequencing were included, resulting in 597 patients in the GPL570 platform cohort and 372 patients in the TCGA-OV cohort. To remove batch effects between RNA-seq and microarray data, we utilized the “sva” package. We also retrieved mutation data from the TCGA-OV cohort, which included 274 samples, and used the “maftools” package to present the top 10 gene mutations. In addition, we downloaded whole-slide digitized histological slides that corresponded to RNA-seq data from the TCGA database.

### 2.2 Cell culture

The HO-8910 and A2780 cell lines as well as human lymphatic tube endothelial cells (HLECs) were obtained from Dr. Feng at Southeast University. HO-8910 and A2780 cells were cultured in RPMI-1640 supplemented with 10% fetal bovine serum (FBS), while HLECs were cultured in Endothelial Cell Medium (ScienCell Research Laboratories, Inc.) with 10% FBS. The cultures were maintained at 37°C in a humidified incubator with 5% CO_2_. To stimulate CXCL10 *in vitro*, we purchased recombinant human CXCL10 from Peprotech and incubated cells with 100 and 200 ng/ml hCXCL10 for 48 h.

### 2.3 Lymphangiogenesis and cell adhesion assay

A precooled 96-well plate was first filled with 50 μL of precooled PBS, followed by the addition of 50 μL Matrigel Basement Membrane (BD Biosciences). HLECs were diluted with conditioned medium, and 60,000 cells were introduced into each well. Lymphangion formation was observed to begin at 6 h and reach completion around 10 h. HO-8910 and A2780 cells were then seeded onto the HLECs in a separate 96-well plate for 30 min, washed twice with PBS to remove free cells, and lysed by the addition of red blood cell lysate for 10 min. The absorbance at 690 nm was measured, and the NC group was normalized to 1.

### 2.4 Single-cell analysis

The Tumor Immune Single-cell Hub (TISCH) is a scRNA-seq database that characterizes the TME at the single-cell level [18]. To explore cell interactions in GSE154600, we used this database. The single-cell level expression matrix was standardized by scaling the raw counts (UMI) in each cell to 10,000 using Seurat’s “NormalizeData” function. The data were then log-transformed. More detailed pre-processing steps are available in the TISCH database documentation function. We focused on CXCL10-related data from the cell–cell interaction module.

### 2.5 Construction and functional verification of molecular subtypes

In the meta-cohort (GPL570 platform and TCGA-OV), univariate cox regression analysis was used to determine the prognostic value of IRRGs, followed by unsupervised consensus clustering to determine the optimal number of clusters (*k* value) using “consensusClusterPlus” package and principal component analysis (PCA) to determine subtype heterogeneity. Importantly, we performed consensus clustering with a range of *k* values (from 2 to 10), and found that *k* = 3 gave the most stable and significant clustering results based on the consensus heatmap and consensus cumulative distribution function plots. Kaplan–Meier analysis and log-rank test were used to assess the different for overall survival (OS). For functional analysis, differential expression genes (DEGs) between subtypes were analyzed using the limma package (adj. *p* < 0.05, |logFC| > 1), and Kyoto Gene and Genome Encyclopedia (KEGG) enrichment analysis was performed using the “clusterProfiler” package. Moreover, gene set variation analysis (GSVA) was used to assess differences in biological pathways between different subtypes. *p*-value <0.05 and *q*-value <0.05 were considered significant enrichment pathways.

### 2.6 Immune cell analysis

Following the pipeline developed in previous studies [12], we used eight algorithms (TIMER, CIBERSORT, QUANTISEQ, MCP-counter, XCELL, EPIC, ESTIMATE, and ssGSEA) to estimate the abundances of immune cells in different risk groups and molecular subtypes.

### 2.7 Construction and validation of machine learning-derived risk score

To create a consensus prognostic model for EC patients, we employed our prior approach [19,20,21]. First, we created a combination of 101 machine learning algorithms based on the traits of ten models, including LASSO, RSF, GBM, Survival-SVM, SuperPC, ridge regression, plsRcox, CoxBoost, StepCox, and Enet. We chose antecedent models with variable filtering capabilities (RSF, CoxBoost, StepCox, and LASSO). Subsequently, we generated signatures in an expression file using predictive IRRGs in the TCGA-OV training cohort. Finally, we calculated risk scores using the obtained signatures in the training cohort and validated the scores in other testing cohorts (GSE19829, GSE18520, GSE9891, GSE26193, GSE30161, and GSE63885) to identify the best consensus prognostic model based on the mean *C*-index of the seven cohorts. We plotted ROC curves to evaluate the predictive accuracy of the risk score and used Cox regression analysis to determine the independent prognostic value of the risk score.

As the cell cycle, PI3K/mTOR pathway, and Wnt pathway have been implicated in AML progression, we used the “pRRophetic” package to calculate the half maximal inhibitory concentration (IC50) of various targeted and chemotherapy drugs, such as CGP.60474 (cell cycle), JW.7.52.1 (PI3K/mTOR), CHIR.99021 (Wnt), cisplatin, bleomycin, and paclitaxel. Relevant references support the role of these pathways in AML progression.

### 2.8 Statistical analysis

Statistical analyses of the genome were conducted using R software (v.4.1.2). Further elaboration on the statistical methods is provided in statistical section in the previous reference [11]. Briefly, to compare categorical variables, we applied the chi-squared or Fisher exact test, while the Wilcoxon rank-sum test or *T*-test was used to compare continuous variables. Statistical significance was defined as *p* < 0.05.

## 3 Results

### 3.1 Identification of inflammatory response-derived subtypes

**Figure 1:**
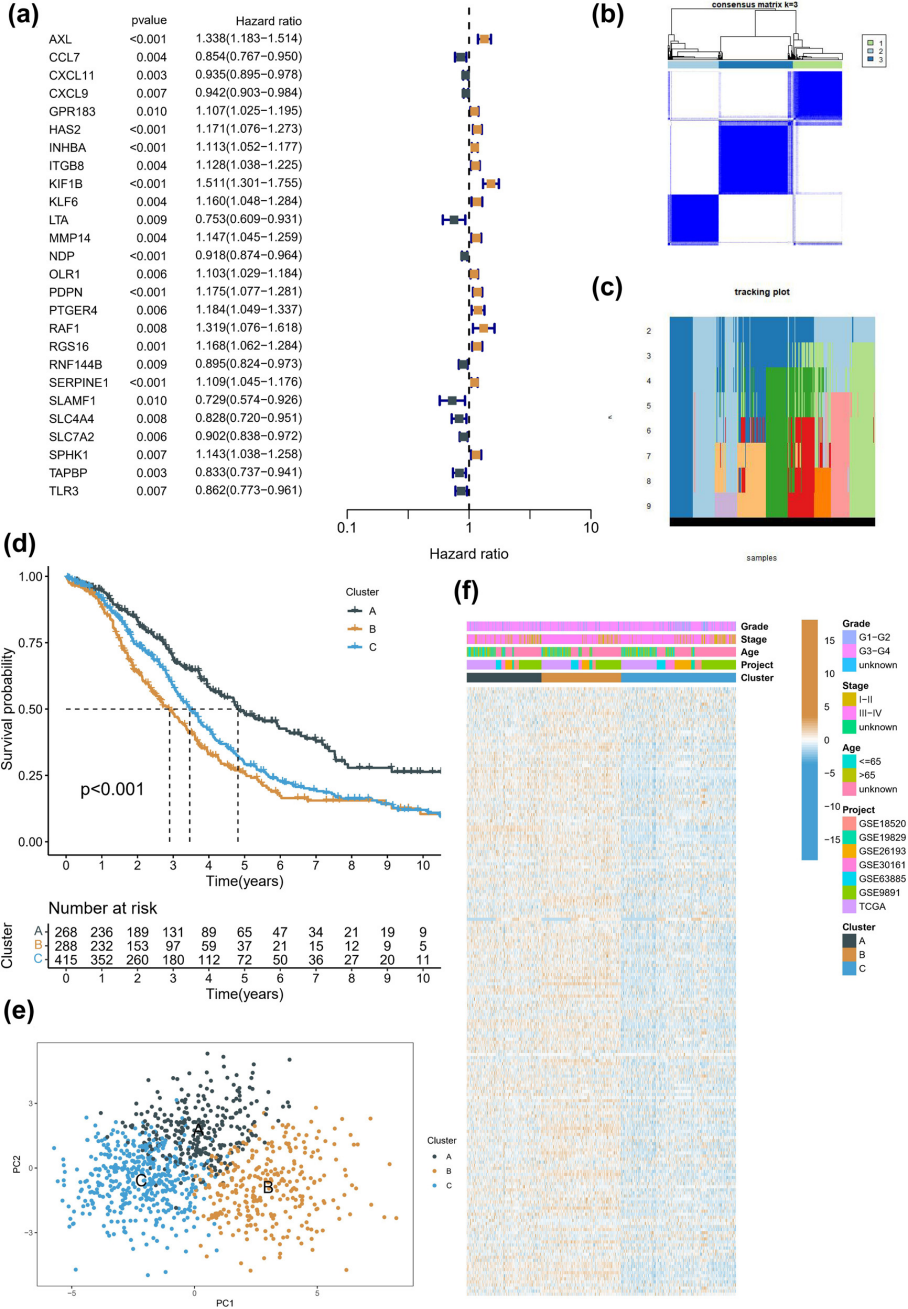
Identification of molecular subtypes associated with inflammatory response. (a) Univariate Cox analysis of IRRGs in the meta cohort. (b and c) Selection of the optimal *k* value based on conensusClusterPlus package. (d) Kaplan-Meier analysis of different molecular subtypes. (e) PCA in three molecular subtypes. (e) A heatmap combined clinical characteristics with gene expression in different molecular subtypes.

In the meta-cohort, we conducted univariate Cox analysis on all IRRGs to identify their prognostic significance. Among them, only 26 IRRGs demonstrated a strong association with survival (Figure 1a). Based on the expression profile of these 26 IRRGs in the meta-cohort, we performed consistent clustering to classify all patients into three molecular subtypes. The clustering results were more stable when *k* = 3 (Figure 1b and c). The three molecular subtypes identified were subtype A, subtype B, and subtype C, with subtype C being the most prevalent (415 patients), and subtypes A and B having similar numbers of patients (268 and 288, respectively). Notably, subtype B had the poorest prognosis, whereas subtype A had the best prognosis (Figure 1d). PCA confirmed the genomic heterogeneity of the different subtypes, with significant dispersion among the three subtypes (Figure 1e). Intriguingly, heatmap analysis of clinical information and IRRGs expression in the meta-cohort revealed that most IRRGs were significantly down-regulated in subtype C (Figure 1f).

### 3.2 Difference in immune microenvironment in different subtypes

**Figure 2:**
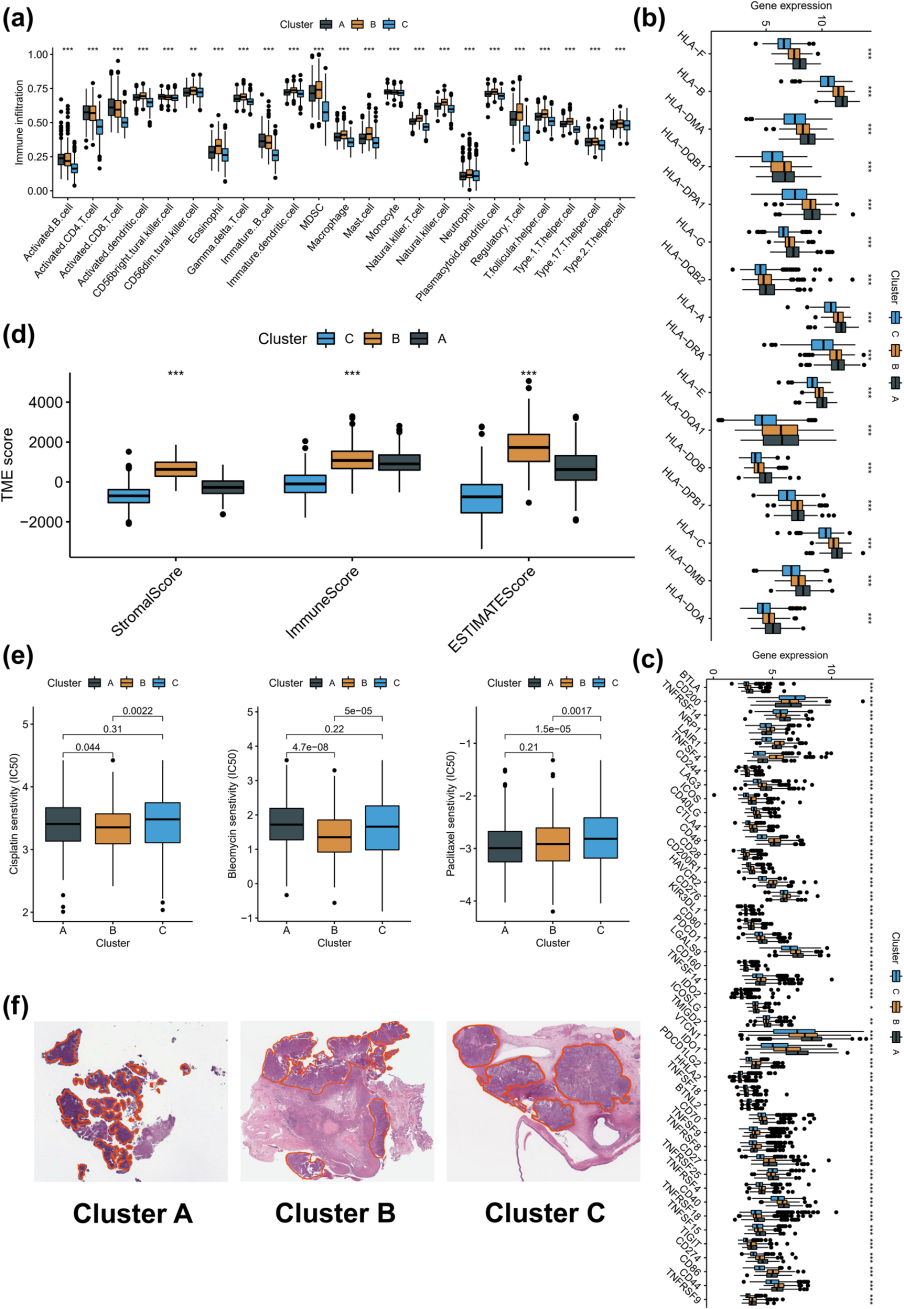
Immune landscape in molecular subtypes associated with inflammatory response. (a) Abundance of 24 immune cells based on ssGSEA algorithm. (b and c) Differences in mRNA associated with ICIs and HLA. (d) Overall TME status based on ESTIMATE algorithm. (e) Differences in the sensitivity of three commonly used chemotherapy regimens (cisplatin, bleomycin, and paclitaxel). (f) HE sections of the different molecular subtypes in the TCGA database. **p < 0.01, ***p < 0.001.

**Figure A1:**
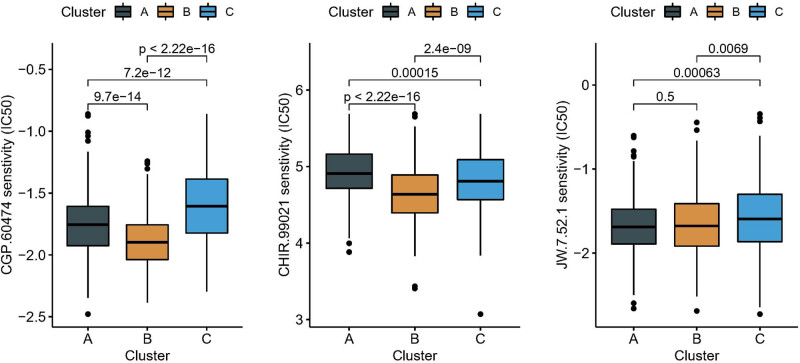
Differences in the sensitivity of three targeted drugs.

The immune microenvironment of different molecular subtypes was analyzed using ssGSEA. Although there was no significant difference in the number of activated T cells between subtype A, which had a better prognosis, and subtype B, which had the worst prognosis, the number of Treg cells was significantly higher in subtype B compared to the other subtypes. These results suggest that subtype C has a greater infiltration of immune cells, but its immune cell function is suppressed, which may be the main reason for its poorer prognosis (Figure 2a). In addition, a comparison of HLA and ICI mRNA expression in different molecular subtypes revealed higher expression in subtype A (Figure 2b and c). The ESTIMATE algorithm was used to re-evaluate the overall TME landscape of different subtypes, and subtype B had the highest stromal score (Figure 2d). To investigate the sensitivity of different subtypes to commonly used chemotherapy regimens (cisplatin, bleomycin, and paclitaxel), we found that subtype B, with more immune-infiltrating cells, had better sensitivity to the three chemotherapeutic agents (Figure 2e). Notably, subtype B was also more sensitive to CHIR.99021, JW.7.52.1, and CGP.60474 (Figure A1). Furthermore, the combination of corresponding HE sections of the molecular subtypes in the TCGA database revealed more lymphoid follicle-like structures in subtype B (Figure 2f).

### 3.3 Difference in functional analysis in different subtypes

**Figure 3:**
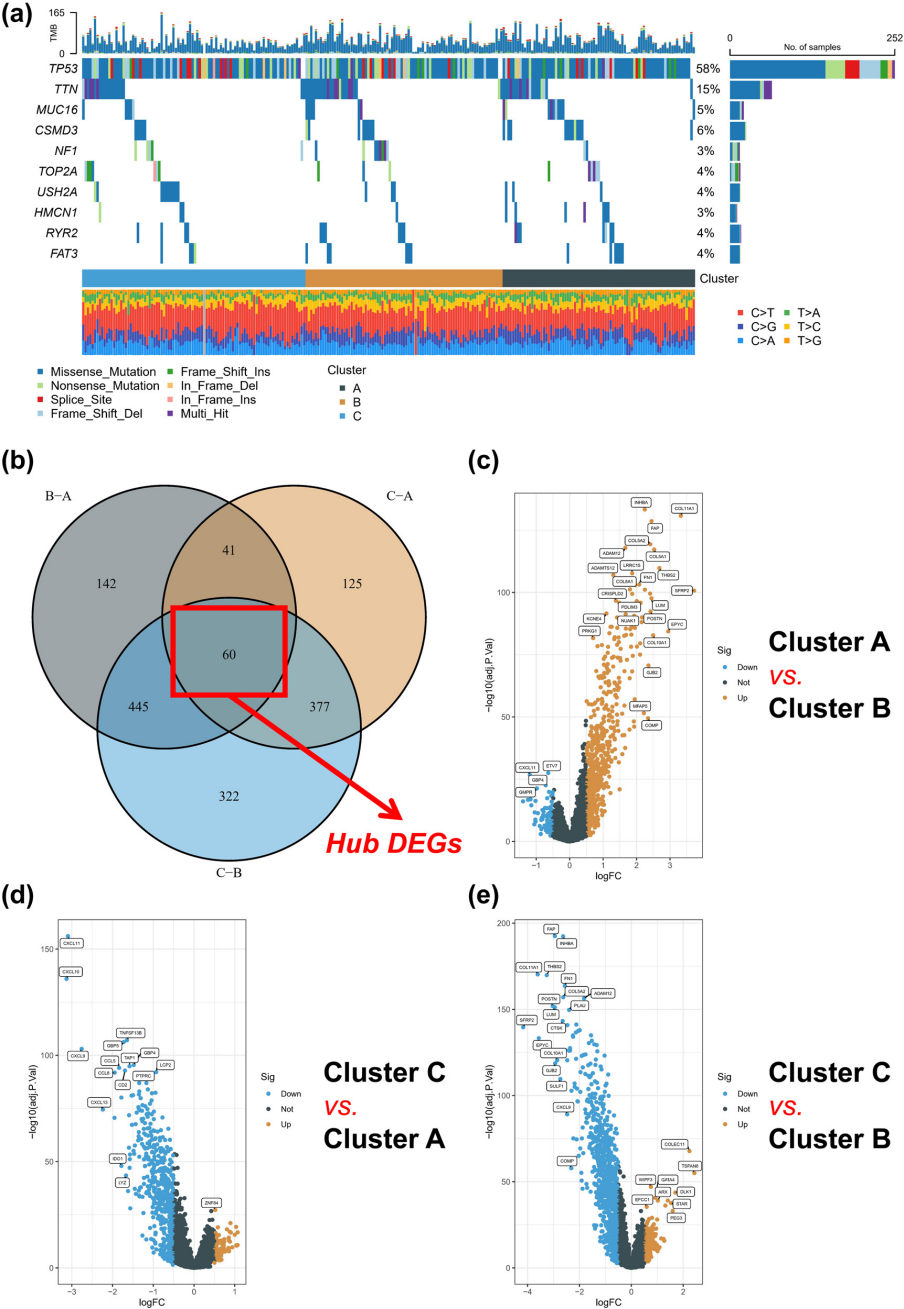
Genetic variants in different molecular subtypes. (a) The top 10 mutated genes in the different molecular subtypes. (b) Common differential expression genes in molecular subtypes. (c) Volcano map of DEGs for subtypes A and B. (d) Volcano map of DEGs for subtypes C and A. (e) Volcano map of DEGs for subtypes C and B.

**Figure 4:**
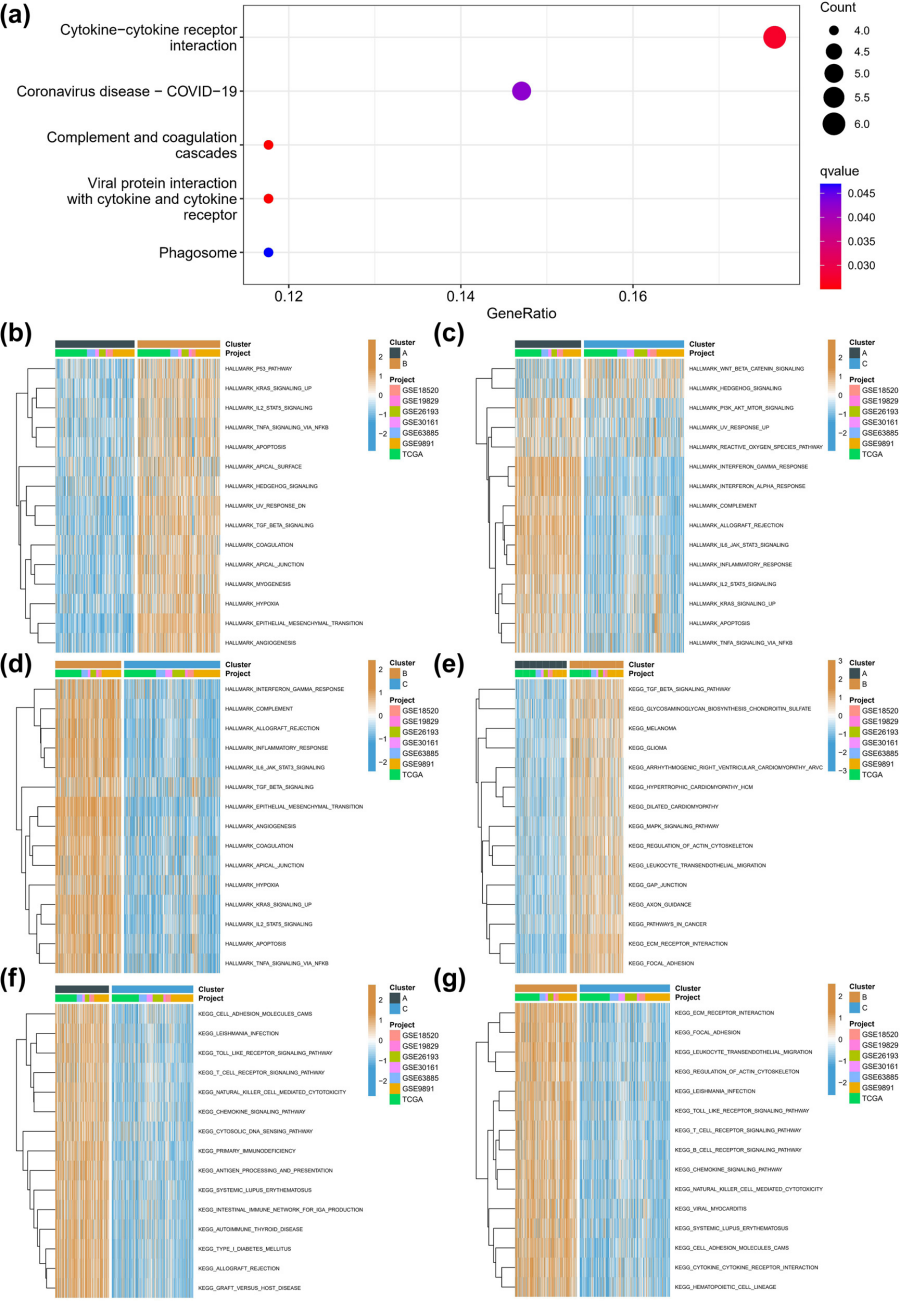
Biological function in molecular subtypes associated with inflammatory response. (a) KEGG enrichment analysis of DEGs. (b) Heatmap of matrix of Hallmark enrichment scores using GSVA algorithm in subtypes A and B. (c) Heatmap of matrix of Hallmark enrichment scores using GSVA algorithm in subtypes A and C. (d) Heatmap of matrix of Hallmark enrichment scores using GSVA algorithm in subtypes B and C. (e) Heatmap of matrix of KEGG enrichment scores using GSVA algorithm in subtypes A and B. (f) Heatmap of matrix of KEGG enrichment scores using GSVA algorithm in subtypes A and C. (g) Heatmap of matrix of KEGG enrichment scores using GSVA algorithm in subtypes B and C.

We performed a comparative analysis of the mutation differences among different molecular subtypes. Remarkably, MUC16 and CSMD3 had significantly higher mutation frequencies in subtype C than in subtypes A and B, which were among the top 10 mutations in the TCGA-OV cohort (Figure 3a). We further identified 60 DEGs between subtypes, with the volcano map highlighting significant changes in the collagen and chemokine families, such as CXCL10, CXCL11, COL11A1, COL5A2, and COL5A1 (Figure 3b–e). KEGG analysis revealed that cytokine–cytokine receptor interaction was a major enrichment pathway of the 60 DEGs (Figure 4a). Based on KEGG and Hallmark gene sets, GSVA showed that compared to subtype A, subtype B was significantly enriched in p53, KRAS signaling, and IL2-STAT5 signaling pathways (Figure 4b). Subtype C was significantly enriched in wnt-beta and hedgehog signaling pathways, while subtype B was enriched in interferon gamma response, complement pathway, and inflammatory response, compared to subtype A (Figure 4c and d). Furthermore, subtype B was enriched in TGF beta signaling pathway, glycosaminoglycan biosynthesis chondroitin sulfate, and melanoma compared to subtype A (Figure 4e), whereas subtype A was significantly enriched in cell adhesion molecules cams, leishmania infection, and toll-like receptor signaling pathway compared to subtype C (Figure 4f). Finally, compared to subtype C, subtype B was enriched in ECM receptor interaction, focal adhesion, and leukocyte transendothelial migration (Figure 4g).

### 3.4 Construction and validation of a machine learning-based signature

**Figure 5:**
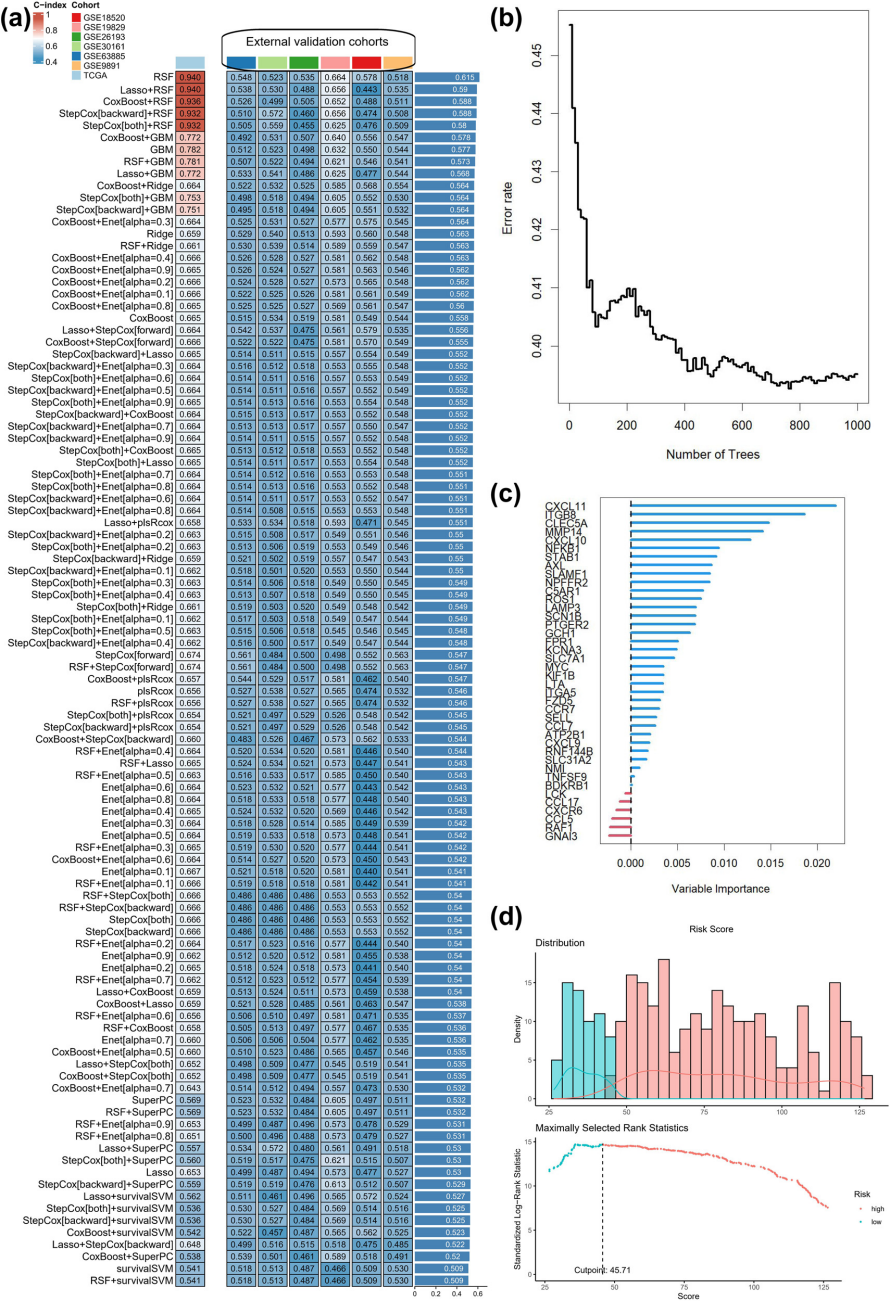
Construction of machine learning-derived signature. (a) The *C*-indexes of 101 machine-learning algorithm combinations. (b) Error rate curve of random forest tree model. (c) Importance of each variable in random forest tree model. (d) Risk score distribution map and optimal cut-off value selection.

**Figure A2:**
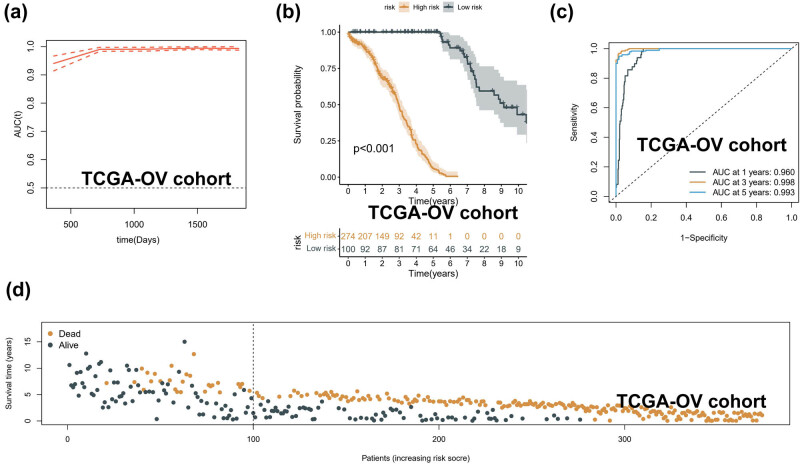
Validation of prognostic signature. (a) The distribution between risk groups and clinical features. (b) The differences in risk scores across clinical subgroups. (c) AUC for the 1-year, 2-year, and 3-year OS in the TCGA-OV cohort. (d) Risk score distribution map.

We conducted an analysis to determine the effectiveness of different algorithms in predicting the risk of OC. Our results showed that the RSF algorithm had the highest average *C*-index (0.615) across all cohorts (Figure 5a and b). The top 5 IRRGs identified by the RSF model were CXCL11, ITGB8, CLEC5A, MMP14, and CXCL10 (Figure 5c). We then established an optimal cut-off value of 45.71 based on risk scores in the TCGA-OV cohort (Figure 5d). Notably, the AUC values for the ROC curve at each time point in the training set exceeded 0.9 (Figure A2a). Additionally, the Kaplan–Meier survival curve revealed that high-risk patients, of the 374 in the training set, had significantly reduced OS (Figure A2b). The AUC values for the 1-, 2-, and 3-year OS were 0.960, 0.998, and 0.993 in the training cohort (Figure A2c). Finally, the risk survival distribution showed that OC patients with a higher risk score had a worse prognosis (Figure A2d).

### 3.5 Evaluating clinical significance of machine learning-derived risk score

**Figure 6:**
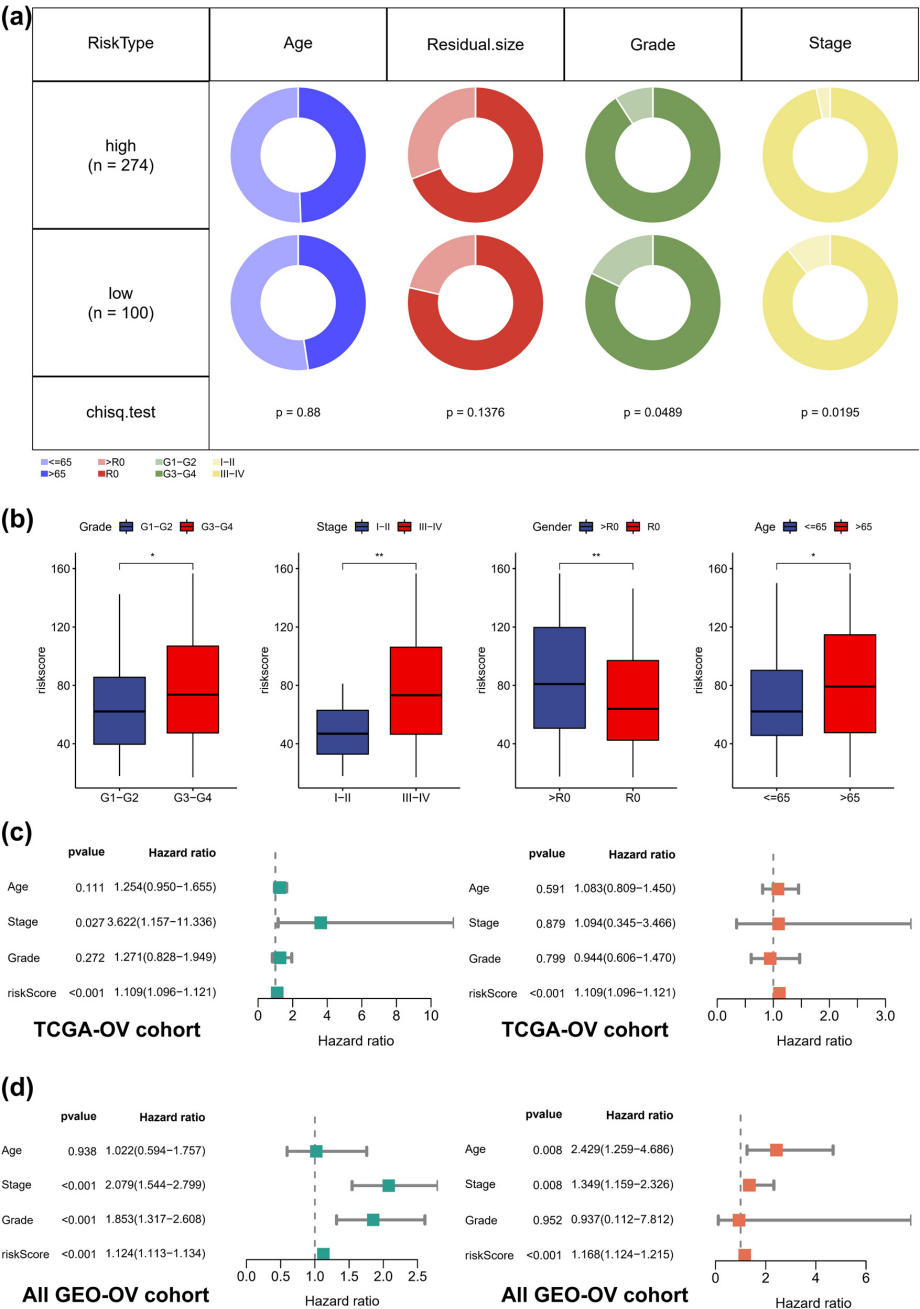
Clinical significance of risk score. (a) The differences in risk scores across clinical subgroups, including age, grade, FIGO stage, and pathologic types. (b) Kaplan–Meier analysis of different age subgroups. (c) Cox regression analyses in TCGA-OV cohorts. (d) Cox regression analyses in GEO-OV cohorts. *p < 0.05, **p < 0.01.

The distribution of FIGO stage was markedly distinct between the high- and low-risk groups (Figure 6a). Notably, our findings indicated a significant association between risk score and advanced age, higher FIGO stage, and higher grade (Figure 6b). Furthermore, we observed that patients with larger postoperative residual size had higher risk scores, suggesting that our risk score could serve as a useful tool for preoperative guidance. Cox regression analyses were performed in both the TCGA-OV (Figure 6c) and all GEO-OV cohorts (Figure 6d), confirming the independent prognostic value of the risk score.

### 3.6 Macrophage-derived CXCL10 is the hub indicator of signature

**Figure 7:**
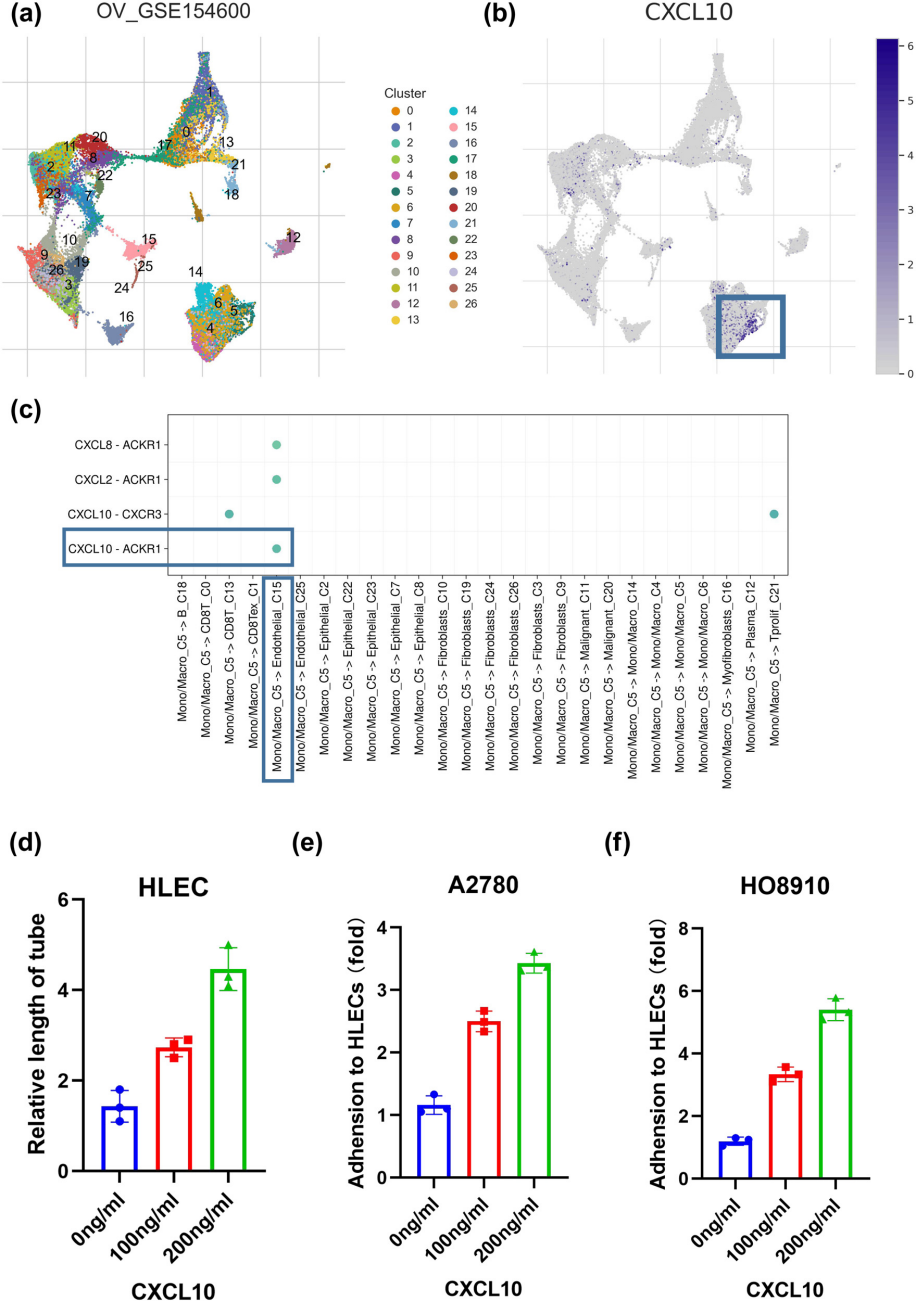
Macrophage-derived CXCL10 in ovarian cancer. (a) Clusters of single-cell dataset. (b) Localization of CXCL10. (c) Cell communication analysis between different cell types. (d) Relative length of tube in different concentration of CXCL10. (e) The ability of adhesion of A2780 to HLECs in different concentration of CXCL10. (f) The ability of adhesion of HO-8910 to HLECs in different concentration of CXCL10.

**Figure A3:**
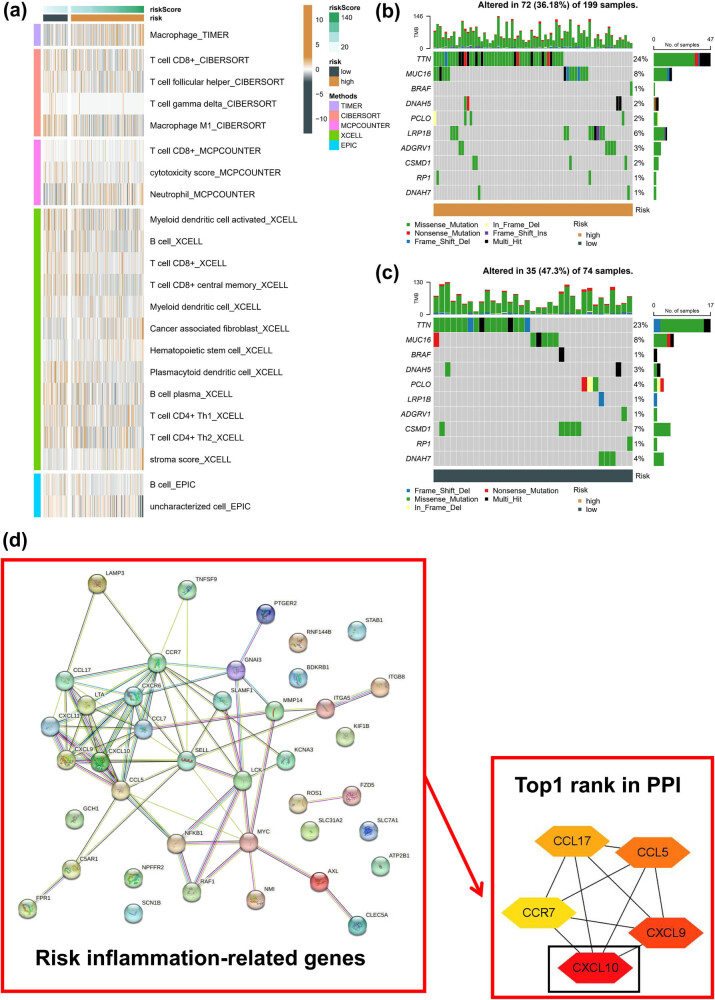
Identification of hub IRRGs in PPI network. (a) Difference of abundance of immune cells based on six algorithms in different risk groups. (b and c) The top 10 mutated genes in the high-risk and low-risk group. (c) PPI network of IRRGs in prognostic model.

In our analysis of immune cell infiltration, we employed multiple algorithms to estimate immune cell activity across various samples. The resulting heatmap indicated that the low-risk group had a more active TME (Figure A3a). Although patients in both the high- and low-risk groups did not exhibit significant differences in top mutated genes based on whole-exome sequencing data, the overall mutation frequency was higher in the low-risk group (Figure A3b and c). To explore the molecular importance of immune-related risk genes (IRRGs) involved in the RSF model, we constructed a protein–protein interaction (PPI) network in the STRING database and ranked the IRRGs by maximum matching coefficient (MMC) (Figure A3d). CCL17, CCR7, CXCL10, CXCL9, and CCL5 were the top 5 genes in the PPI network, with CXCL10 being the most important. Using single-cell data, we confirmed that CXCL10 is mainly located in macrophages (C5) (Figure 7a). Cell communication analysis indicated a significant association of CXCL10 between macrophages and endothelial cells (C15) (Figure 7b–c). LYVE1, the lymph endothelial cell marker, was significantly overexpressed in the C15 subgroup (data not shown). We, therefore, hypothesize that CXCL10 secreted by macrophages promotes lymphangiogenesis and cell adhesion. As anticipated, with increasing hCXCL10 concentrations, lymphatic tube growth was observed (Figure 7d), along with increased adhesion to tumor cells (Figure 7e and f).

## 4 Discussion

Chronic inflammation plays a critical role in cancer, and OC’s TME exhibits elevated levels of inflammatory proteins [22]. TCGA has described an immune-responsive subtype of high-grade plasmacytosis characterized by the expression of T-cell chemokine ligands CXCL11 and CXCL10 and the receptor CXCR3 and observed overexpression of inflammatory lipoxygenase pathway receptors [23]. A recent study found that minor alleles ALOX5 in rs17561 and rs4848300 and rs1864414 in IL-1A were consistently and negatively associated with the risk of OC [24]. However, no study has investigated genetic signatures associated with inflammatory response, TME, and drug sensitivity in OC. Therefore, in our study, we performed consistent clustering to classify all patients based on IRRGs. We identified three molecular subtypes, with subtype C accounting for the majority of all patients, and subtype B exhibiting the worst prognosis.

We observed that while the number of activated T cells did not differ significantly between subtype A, which had a favorable prognosis, and subtype B, which had the worst prognosis, the number of Treg cells was significantly higher in subtype B than in other subtypes. Our findings suggest that subtype C has a higher infiltration of immune cells, but the function of its immune cells is suppressed, which may contribute to its poorer prognosis. Regulatory T cells, which are tumor immunosuppressors, have emerged as a critical area of research to identify the underlying biological mechanisms associated with OC development and progression [25]. Although Treg cells are known to inhibit CD4+ and CD8+ T cells [26,27], evidence also suggests that they suppress natural killer cells and specialized antigen-presenting cells, such as dendritic cells [28]. Furthermore, in an intentional epidemiological survey, the average frequency of Treg cells was significantly higher in newly diagnosed OC patients than in women with benign ovarian disease and cancer-free controls [27].

Our study has revealed that the B subtype, characterized by more immune-infiltrating cells, displayed better sensitivity to the three chemotherapeutic agents. Further analysis of the corresponding HE sections of the molecular subtypes revealed more lymphoid follicle-like structures in the B subtype. The known effects of chemotherapy on the TME of OC include enhanced T-cell activation, increased density of T cells, B cells, and NK cells and decreased density of Treg cells [29,30,31]. Our findings suggest that our inflammatory response-related subtypes may be associated with chemotherapy sensitivity and underscore the critical role of the TME.

We computed the average *C*-index for all cohorts to evaluate the machine learning-based signature. The RSF algorithm yielded the highest *C*-index. The top 5 IRRGs of importance in the RSF model were CXCL11, ITGB8, CLEC5A, MMP14, and CXCL10. Notably, the AUC value at each time point in the training set was above 0.9. Previous studies have constructed prognostic signatures for OC based on cancer-associated fibroblasts [11] or ferroptosis-related long non-coding RNA [32], using only the LASSO-Cox modeling scheme. To avoid personal preferences and inappropriate modeling approaches, we combined 101 machine learning algorithms and selected the best-performing model. We examined the molecular significance of the IRRGs identified by the RSF model by constructing a PPI network in the STRING database. The top 5 genes in the PPI network were CCL17, CCR7, CXCL10, CXCL9, and CCL5, with CXCL10 being the most critical. Notably, our study is the first to investigate the correlation of CXCL10 with human lymphatic endothelial cells. An earlier study highlights the clinical relevance of the CXCL10 + IRF1 + STAT1 + macrophage subset as a biomarker for intratumoral T-cell activation [33]. A combined bioinformatics *in vitro* assay suggested that CXCL10 could impact OC progression by increasing the expression of cytotoxic T cells and inhibiting angiogenesis [34].

However, our study has some limitations that need to be acknowledged. First, further experiments are required to unravel the mechanism underlying CXCL10’s impact on human lymphatic endothelial cells. Second, although we validated our findings on six external cohorts, prospective clinical trials involving larger local cohorts are necessary. In conclusion, our machine learning-based signature for the inflammatory response can distinguish the TME and predict prognosis in patients with OC.

## 5 Conclusion

The study aimed to investigate the role of IRRGs in OC and develop a machine learning-based inflammatory response-related signature to identify distinct TME and predict patient prognosis. The study integrated mRNA expression profiles from seven cohorts and identified CXCL10 as a critical factor in distinguishing patient prognosis based on the expression of prognostic IRRGs. The study also confirmed the distribution ratios of stromal cells, inflammatory cells, and tumor cells using whole-slide digitized histological slides and elucidated differences in biological pathway activation among subtypes using KEGG-related gene sets. Single-cell and *in vitro* experiments confirmed that macrophage-derived CXCL10 promotes lymphangiogenesis and cell adhesion. Overall, the study provides new insights into the role of IRRGs in OC and may have important implications for the development of novel therapeutic approaches.

## Appendix

**Table A1:** Detailed description for different datasets

Dataset	Platform	Samples	Type
GSE19829	Affymetrix Human Genome U133 Plus 2.0 Array	28	Microarray
GSE30161	Affymetrix Human Genome U133 Plus 2.0 Array	58	Microarray
GSE63885	Affymetrix Human Genome U133 Plus 2.0 Array	75	Microarray
GSE26193	Affymetrix Human Genome U133 Plus 2.0 Array	107	Microarray
GSE9891	Affymetrix Human Genome U133 Plus 2.0 Array	276	Microarray
GSE18520	Affymetrix Human Genome U133 Plus 2.0 Array	53	Microarray
TCGA_OV	Illumina HiSeq 2000	374	RNA-seq
